# Mental illness and non-metastatic colorectal cancer treatment and survival, a nationwide study of almost 70,000 patients

**DOI:** 10.2340/1651-226X.2025.42710

**Published:** 2025-04-30

**Authors:** Erik Osterman, Elisavet Syriopoulou, Anna Martling, Therese M.-L. Andersson, Caroline Nordenvall

**Affiliations:** a Department of Molecular Medicine and Surgery, Karolinska Institute, Stockholm, Sweden; b Department of Surgical Sciences, Uppsala University and Department of Surgery, Uppsala University Hospital, Uppsala, Sweden; c Department of Medical Epidemiology and Biostatistics, Karolinska Institute, Stockholm, Sweden; d Department of Pelvic Cancer, Colorectal Surgery Unit, Karolinska University Hospital, Stockholm, Sweden

**Keywords:** Disparities, colorectal cancer, flexible parametric models, treatment, survival, time to recurrence, survival after recurrence

## Abstract

**Background and purpose:**

The impact of mental illness on treatment and outcomes for patients with colorectal cancer (CRC) has not been investigated with potential confounders and mediators accounted for.

**Patients and methods:**

Colorectal Cancer Database (CRCBaSe), a linked national registry database, was used to analyse stage I–III CRC patients diagnosed in Sweden between 2008 and 2021. The exposure of interest was a history of mental illness. Treatment outcomes were analysed with logistic regressions. Flexible parametric models were fitted for survival analysis. Analyses were adjusted for pre-specified confounders.

**Results:**

Patients with a history of severe mental illness presented with more advanced tumours and comorbidities. They were more likely to undergo emergency surgery (OR 1.56, 95% CI 1.32–1.84) and less likely to receive adjuvant treatment (OR 0.65, 95% CI 0.53–0.80) than patients with no history of mental illness. Five-year standardised overall survival (OS) was worse for those with a history of mild and severe mental illness, 64.6% (95%CI 63.9–65.3) and 61.8% (95%CI 59.7–63.8) compared to those without 69.3% (95%CI 68.9–69.7). Although time to recurrence was not significantly impacted, standardised survival after recurrence was worse for patients with a history of severe mental illness, with a 3-year survival after recurrence of 24% compared to 30% in those without a history of mental illness.

**Interpretation:**

Although the differences were smaller compared to previous studies, patients with a history of mental illnesses still do worse. The management of CRC patients with psychiatric comorbidities presents complex challenges necessitating personalised solutions.

## Introduction

Persons with severe mental illness (bipolar, depressive disorders and psychotic disease) who develop colorectal cancer (CRC) have been shown to have more severe disease at diagnosis [1], to be less likely to receive surgery and adjuvant therapy [2–6] and to have worse cancer-specific survival (CSS) [1]. Patients with severe mental illness are often socioeconomically deprived [7, 8]. Socioeconomic status (SES) impacts the risk of emergency surgery and the most deprived have lower odds of receiving neoadjuvant and adjuvant therapy [9], and have worse overall and CSS [10]. Previous results of impaired prognosis following CRC diagnosis in patients with mental illness could, at least partly, be explained by factors related to SES. The disparities are observed in many different healthcare systems, including ones with universal access like the Swedish [3, 11]. Previous studies have been limited by size, selection and detailed data of potential confounders including data on socioeconomic factors [12, 13].

This study aimed to investigate if there are differences in treatment and outcomes of stage I–III CRC in patients with a history of mental illness in Sweden using population-based data. The hypothesis was that patients with a history of mental illness have more advanced tumours, are less likely to receive oncological treatment, are more likely to undergo emergency cancer resection and have worse survival and more recurrences.

## Patients and methods

Patient data originated from the Colorectal Cancer Database (CRCBaSe), a register-linkage of the Swedish Colorectal Cancer Registry (SCRCR) and national registries at the National Board of Welfare and Statistics Sweden described previously [14]. All adults (≥18 years old) with a stage I–III CRC in CRCBaSe with a first-time diagnosis between 2008 and 2021 were included in the study. Patients with synchronous metastatic disease or stage 0 were excluded. In cases of multiple tumours, the cancer was classified according to the ‘worst’ tumour (highest T and N stage). Patients who had surgery but partially missing data on the TNM stage were classified as stage III if TxN1-2M0, stage II if TxN0M0 and T3-4NxMx and stage I if T1-2NxMx.

### Exposure

History of mental illness was classified according to the patient registry covering psychiatric inpatient care since 1974 (complete coverage since 1987), psychiatric out-patient care since 2001 and the prescription registry covering all prescriptions since July 2005. Healthcare contacts with an International Classification of Disease 10 code (ICD10 used since 1997) of F20–F22, and F30–F39 which are the Swedish codes for schizophrenic disorders, psychosis, bipolar disorders and depressive disorders were used to identify patients with a history of mental illness before CRC diagnosis. Prescription of medications before CRC diagnosis with Anatomical Therapeutic Chemical Classification System (ATC) codes: N06A, N06B and N06C, which are anti-depressives, were used to find patients with a history of mental illness without in-patient or out-patient contact with specialised psychiatric care. Patients with a diagnosis from the patient registry were classified as having a severe mental illness as they required specialised care, while patients with only an anti-depressive medication and no codes for psychiatric diagnoses from the patient registry were classified as having a mild mental illness as they did not require specialised care. Patients without codes or prescriptions were classified as having no mental illness.

### Outcomes

The following treatment outcomes were collected from the SCRCR and examined:

1. Surgical treatment (cancer resection)
2. Neoadjuvant treatment (elective rectal cancer patients)
3. Emergency cancer resection (in all resected patients)
4. Multi-disciplinary team rounds before surgery (elective patients)
5. Multi-disciplinary team rounds after surgery (patients alive 30 days after surgery)
6. Adjuvant treatment (colon cancer patients alive 30 days after surgery)

Survival outcomes were overall survival (OS) in all patients with stage I–III disease, and in those who had surgery the outcomes of interest were: OS, CSS, time to recurrence (TTR) and survival after recurrence (SAR). CSS and TTR was calculated from surgery to death/recurrence. TTR follow-up ended 6 years after diagnosis since the mandatory reporting of recurrences to the registry stops at 5 years (with some delay).

### Other covariates

Socioeconomic status was defined based on two indicators registered by Statistics Sweden: the individual part of disposable household income and the highest education achieved. The average individual part of disposable household income 2 years before diagnosis was split into sex and age-adjusted (±65) quartiles to allocate patients in groups of high and low socioeconomic positions with Q1 being the most deprived and Q4 the least deprived. The highest level of education attained at diagnosis was categorised as <9 years, 9–12 years and >12 years. Civil status at diagnosis was classified into two categories using civil status data and individual and household income data (living alone or not) and included as a covariate in the models. Comorbidities were measured using the Charlson Comorbidity Index (CCI) [15] calculated from the National Patient Registry before cancer diagnosis [16], and The American Society of Anesthesiologists physical status classification (ASA) reported in the SCRCR [17]. Compared to the CCI the ASA classification captures functional limitations instead of relying solely on previous diagnoses. Data on tumour location and pT and pN stage was defined from the SCRCR and included as confounders in the models.

## Statistical methods

Complete case analysis was used since there were few cases with missing data for the exposures and outcomes. The missing data in this setting would be challenging to impute, for example imputing the education status of individuals with missing data based on the observed data requires strong assumptions, this could lead to biased imputed values and potential misclassification in education groups and, consequently, biased estimates of interest. Differences between groups were analysed with the Χ^2^ test and Kruskal–Wallis test.

Logistic regression models were used to assess the effect of mental illness on the treatment outcomes while adjusting for confounders. Direct acyclic graphs were used to assess potential confounders (Supplementary Figure 1). All regression models were adjusted for income, education, year of diagnosis (2008–2021), age at diagnosis (years), sex (male/female), civil status (partner/no), ASA (levels 1–5) and CCI (continuous variable), tumour location (colon/rectum) and stage (pT, pN).

Flexible parametric survival models on the log cumulative hazard scale with four knots for the baseline hazard shape were used to analyse time-to-event outcomes [18, 19]. Knots were placed at equally distributed quantiles of the log of the event times. Three flexible parametric survival models were fitted for each outcome: (1) no exposure, (2) with the exposure and (3) with a time-varying effect for the exposure. All these models were adjusted for the variables described previously. Models were compared using the likelihood ratio test and the best model was used to obtain standardised survival curves and hazard ratios (HR).

Survival estimates and HR for OS, CSS, TTR and SAR were obtained from the models. To compare survival estimates across groups of mental illnesses accounting for differences in the characteristics between groups, standardised survival was estimated. Standardised survival was obtained by estimating the individual-specific survival probabilities given each individual’s covariate pattern except for the group with mental illness and then averaging them. This was done using the covariate pattern seen in the patients with severe mental illness and changing the mental illness classification to no or mild mental illness. These estimates are adjusted for all variables included in the flexible parametric survival models. They can be interpreted as the survival of the patients with severe mental illness and the survival of the same patients if their mental illness status could be moved to the other mental illness status groups (keeping their covariate pattern for all the other covariates unchanged).

OS, CSS and TTR models were adjusted for income, education, civil status, sex, age (using restricted cubic splines to allow non-linear effects with three knots at 56, 73, 85), year of diagnosis, ASA, CCI, T and N stage and tumour location. For SAR, adjustments for income, education, civil status, year of diagnosis, sex, age (knots at 55, 71, 83) and CCI were used. When analysing OS for all patients, including those not having surgery, ASA was not used for standardisation. HR and standardised survival proportions were calculated at 1, 3, and 5 years. Sensitivity analysis was done by fitting additional OS, CSS and TTR models in the resected cohort, adjusted for statistically significant treatment outcomes that could be mediators to assess how these treatment differences affect the survival outcomes. Survival estimates were then obtained similarly to the main analysis but this time standardising over these treatment variables.

*P*-values <0.05 were considered statistically significant. Statistics were calculated using R (version 4.3.2; R Core Team 2023) and the rstpm2 package [20, 21] was used to produce flexible parametric survival models and calculate HR, rates and survival curves. The Regional Board of the Ethical Committee in Stockholm and the National Ethical Committee approved the study. The article was prepared following the STROBE guidelines.

## Results

**Figure 1:**
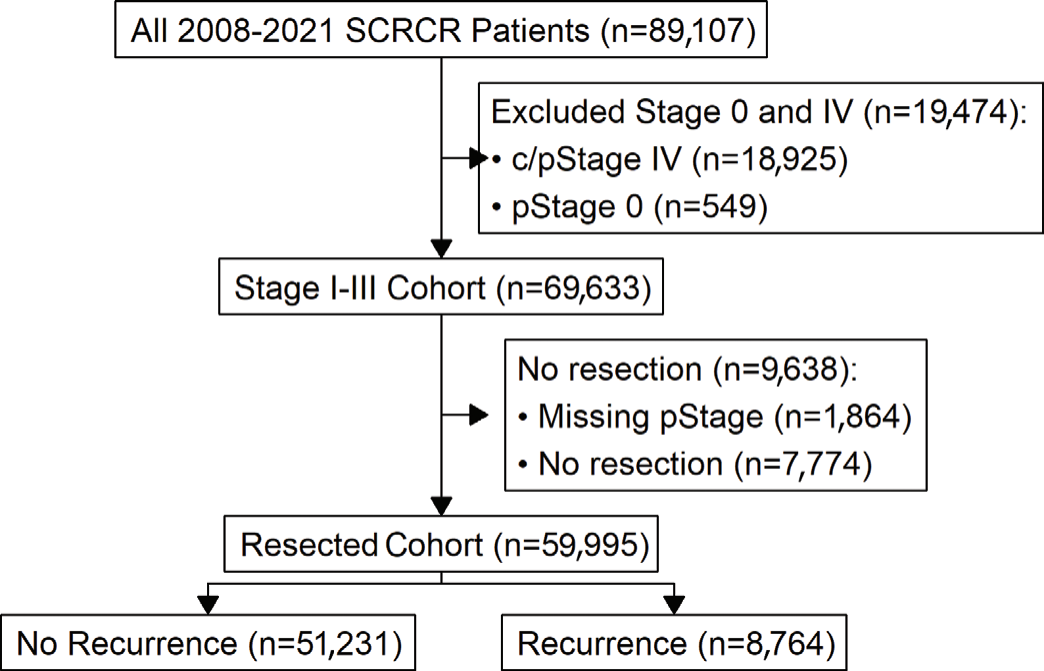
Selection of three cohorts; (1) all Stage I–III patients diagnosed with colorectal cancer (CRC) between 2008 and 2021, (2) all patients with final pathological stage and resection for stage I–III CRC and (3) patients with recurrence after surgery for stage I–III CRC.

There were 89,107 patients diagnosed with first-time CRC between 2008 and 2021 included in the database. After the exclusion of patients with synchronous metastatic disease or stage 0 there were 69,633 patients diagnosed with stage I–III CRC (Figure 1). After exclusion of patients where no resection was performed or who lacked final pathological stage there were 59,995 patients in the resected cohort, described in Table 1.

**Table 1:** Demographics of resected colorectal cancer patients diagnosed with stage I–III disease in Sweden during the years 2008–2021 by the history of mental illness (no, mild, severe mental illness).

Variables	Total (%)	No (%)	Mild mental illness (%)	Severe mental illness (%)
Sex				
Male	31,298 (52.2)	26,012 (55.4)	4,611 (39.1)	675 (54.0)
Female	28,697 (47.8)	20,927 (44.6)	7,195 (60.9)	575 (46.0)
Age (Years)				
Median (IQR)	73.0 (65.0–80.0)	73.0 (65.0–79.0)	73.0 (65.0–80.0)	72.0 (64.0–79.0)
ASA				
1	8,280 (13.8)	7,193 (15.3)	986 (8.4)	101 (8.1)
2	31,114 (51.9)	24,687 (52.6)	5,842 (49.5)	585 (46.8)
3	17,741 (29.6)	12,955 (27.6)	4,305 (36.5)	481 (38.5)
4	1,607 (2.7)	1,071 (2.3)	478 (4.0)	58 (4.6)
5	31 (0.1)	27 (0.1)	4 (0.0)	0 (0.0)
Missing	1,222 (2.0)	1,006 (2.1)	191 (1.6)	25 (2.0)
CCI				
Median (IQR)	0.0 (0.0–2.0)	0.0 (0.0–2.0)	0.0 (0.0–2.0)	0.0 (0.0–2.0)
Missing	3,727 (6.2)	3,727 (7.9)	0 (0)	0 (0)
Income				
Q1	13,482 (22.5)	10,033 (21.4)	3,080 (26.1)	369 (29.5)
Q2	13,914 (23.2)	10,454 (22.3)	3,148 (26.7)	312 (25.0)
Q3	14,327 (23.9)	11,123 (23.7)	2,922 (24.8)	282 (22.6)
Q4	14,481 (24.1)	11,543 (24.6)	2,651 (22.5)	287 (23.0)
Missing	3,791 (6.3)	3,786 (8.1)	5 (0.0)	0 (0.0)
Education				
-9y	14,609 (24.4)	11,350 (24.2)	2,969 (25.1)	290 (23.2)
9y-12y	20,879 (34.8)	15,564 (33.2)	4,799 (40.6)	516 (41.3)
12y-	20,096 (33.5)	15,739 (33.5)	3,925 (33.2)	432 (34.6)
Missing	4,411 (7.4)	4,286 (9.1)	113 (1.0)	12 (1.0)
Civil				
Alone	25,888 (43.2)	19,865 (42.3)	5,428 (46.0)	595 (47.6)
Not alone	34,107 (56.8)	27,074 (57.7)	6,378 (54.0)	655 (52.4)
Mental Illness				
None	46,939 (78.2)	46,939 (100.0)	0 (0.0)	0 (0.0)
Mild depression	11,806 (19.7)	0 (0.0)	11,806 (100.0)	0 (0.0)
Severe depression	16 (0.0)	0 (0.0)	0 (0.0)	16 (1.3)
Schizophrenia/ Psychosis	303 (0.5)	0 (0.0)	0 (0.0)	303 (24.2)
Bipolar disorder	931 (1.6)	0 (0.0)	0 (0.0)	931 (74.5)

ASA: American Society of Anesthesiologists classification; CCI: Charlson Comorbidity Index; IQR: Interquartile range; Q: Quartile.

In the entire cohort comprising all 69,633 stage I–III patients, 53,999 (77.5%) had no mental illness, 14,164 (20.3%) patients had mild mental illness, that is diagnosis of mild depression or prescription of antidepressive or anti-anxiety medication in the absence of a diagnosis of severe mental illness and 1,470 (2.1%) had severe mental illness. In the resection cohort of 59,995 patients, there were 46,939 (78.2%) patients with no mental illness and 11,806 (19.7%) with mild mental illness.

## Demographics

Patient characteristics are presented in Table 1 and Supplementary Table 1 for the resection cohort and the Stage I–III cohort in Supplementary Table 2. Patients with mild mental illness were more likely to be women, more comorbid, had a lower income, fewer years of education, more likely to live alone, have colon cancer and T4 stage compared to patients without mental illness. Patients with severe mental illness were more comorbid than those with no mental illness. They also had a lower income and lived alone to a greater extent than those with no mental illness. In addition, they were more likely to have colon cancer, T4 stage and node positive tumours.

## Differences in treatment

**Figure 2:**
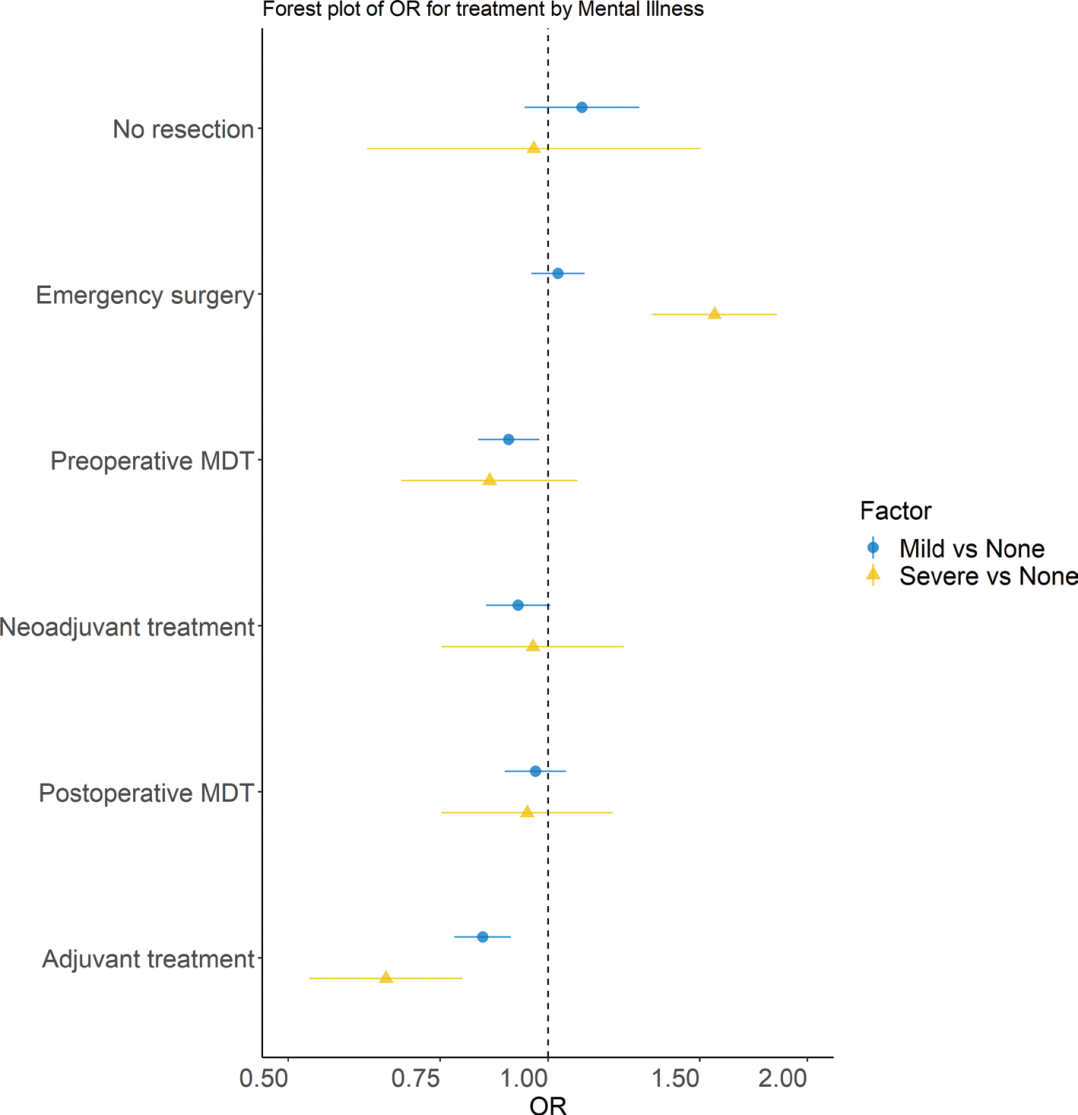
Forest plot including the multivariable ORs obtained from the logistic regression models for treatment outcomes by the history of mental illness. Adjustments were made for income, education, year of diagnosis, age at diagnosis, sex, civil status, ASA, CCI, tumour location and stage. The bar is the 95% confidence interval.

There was no statistically significant difference in the odds of having cancer resection in the stage I–III cohort (Figure 2, Supplementary Table 3). There was no statistically significant difference in the odds of receiving neoadjuvant treatment in the resection cohort. Patients with severe mental illness had higher odds of having emergency surgery than those with no mental illness (OR 1.56 95%CI 1.32–1.84). Among patients undergoing elective cancer resection, preoperative multi-disciplinary team discussions were less common for patients with a history of mild mental illness in comparison to those with none (OR 0.90 95% 0.83–0.98). There was no statistically significant difference by mental illness history in the odds of being discussed at a postoperative multi-disciplinary team discussion if alive 30 days after surgery. History of mental illness was associated with a decreased probability of undergoing adjuvant treatment (mild mental illness: OR 0.84 95%CI 0.78–0.91, severe mental illness: OR 0.65 95%CI 0.53–0.80).

## Survival differences

Standardised survival estimates were obtained using the covariate distribution (i.e. adjusting for income, education, civil status, sex, age, year of diagnosis, comorbidities, T, N and tumour location) of the patients with a history of severe mental illness. The OS and CSS models with a time-varying effect for mental illness were significantly better than those without (likelihood test values in Supplementary Table 4). Adding mental illness to the model for TTR did not improve T.

**Figure 3:**
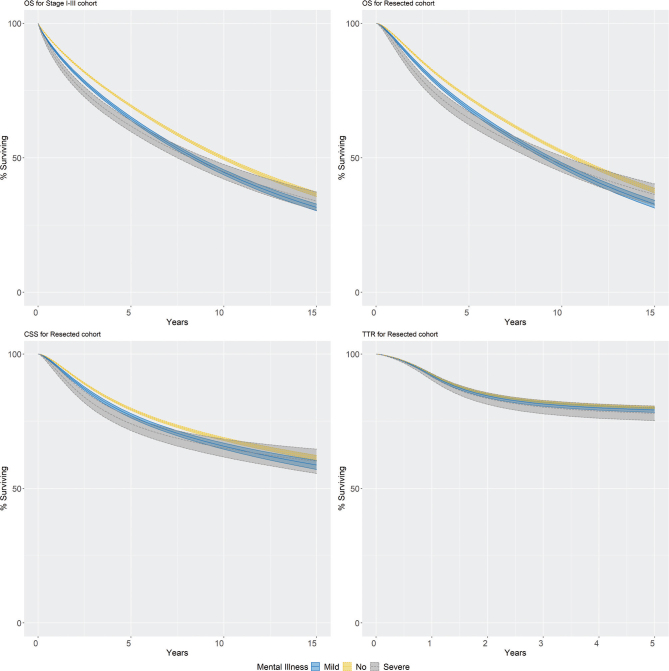
Standardised survival outcomes for non-metastasized colorectal cancer patients by the history of mental illness. Upper left panel: Overall survival in the stage I–III cohort. Upper right panel: Overall survival in the resection cohort. Lower left panel: Cancer-specific survival in the resection cohort. Lower right panel: Time to recurrence in the resection cohort. Standardised to the distribution of income, education, civil status, year of diagnosis, sex, age, CCI, ASA, T and N stage, and tumour location of the patients with a history of severe mental illness. The shaded area corresponds to 95% confidence interval.

In the stage I–III cohort, the standardised 5-year OS were 65 and 62% for those with mild and severe mental illness compared to 69% without mental illness. The HRs for OS comparing mild and severe mental disease to no mental disease were significantly increased at 1 and 3 years but decreased over time (Table 2, Figure 3). Median follow-up until censoring or death was 4.4 years (IQR 1.92–8.1).

**Table 2:** Proportion surviving and Hazard ratios of Overall survival for all (OS all) and Overall survival in resected cohort (OS op), Cancer-specific survival (CSS), Time to recurrence (TTR) and survival after recurrence (SAR) by the history of mental illness (no, mild, severe mental illness) at 1, 3 and 5 years for resected colorectal cancer patients diagnosed with stage I–III disease in Sweden during the years 2008–2021.

Outcome	Mental illness	1-year		3-year		5-year	
		Survival % (95%CI)	HR (95%CI)	Survival % (95%CI)	HR (95%CI)	Survival % (95%CI)	HR (95%CI)
OS all	No	91.4 (91.2–91.7)		79.1 (78.8–79.5)		69.3 (68.9–69.7)	
	Mild	89.1 (88.6–89.5)	1.19 (1.14–1.24)	75.1 (74.5–75.8)	1.14 (1.1–1.17)	64.6 (63.9–65.3)	1.12 (1.07–1.17)
	Severe	86.4 (84.9–87.8)	1.45 (1.31–1.6)	71.5 (69.5–73.4)	1.11 (1–1.23)	61.8 (59.7–63.8)	0.98 (0.83–1.13)
OS op	No	95.4 (95.2–95.6)		82.6 (82.3–83.0)		72.3 (71.8–72.7)	
	Mild	94.0 (93.6–94.4)	1.20 (1.14–1.27)	79.5 (78.8–80.2)	1.13 (1.08–1.17)	68.2 (67.4–69.0)	1.12 (1.08–1.17)
	Severe	92.0 (90.6–93.4)	1.60 (1.40–1.81)	75.1 (72.9–77.4)	1.17 (1.03–1.3)	64.6 (62.2–67.1)	0.95 (0.79–1.12)
CSS	No	96.5 (96.3–96.7)		86.8 (86.4–87.2)		79.5 (79.1–79.9)	
	Mild	95.5 (95.1–95.9)	1.17 (1.09–1.25)	84.7 (84.0–85.4)	1.07 (1.01–1.13)	77.1 (76.3–77.9)	1.05 (0.98–1.12)
	Severe	93.9 (92.6–95.2)	1.53 (1.28–1.79)	81.4 (79.2–83.6)	1.11 (0.93–1.29)	74.1 (71.5–76.6)	0.89 (0.63–1.15)
TTR	No	92.5 (92.2–92.7)		82.3 (81.8–82.7)		80.1 (79.7–80.6)	
	Mild	92.1 (91.6–92.5)	1.05 (1.00–1.11)	81.4 (80.6–82.2)	1.05 (1.00–1.10)	79.2 (78.3–80.1)	1.05 (1.00–1.10)
	Severe	91.5 (90.3–92.7)	1.13 (0.97–1.29)	80.3 (77.8–82.8)	1.11 (0.97–1.25)	78.0 (75.2–80.7)	1.11 (0.97–1.25)
SAR	No	59.0 (57.9–60.2)		29.5 (28.5–30.6)		20.1 (19.2–21.0)	
	Mild	56.9 (55.1–58.7)	1.06 (1.01–1.11)	27.3 (25.6–29.1)	1.06 (1.01–1.11)	18.2 (16.7–19.8)	1.05 (1.01–1.10)
	Severe	50.8 (45.9–55.6)	1.24 (1.09–1.39)	21.5 (17.2–25.8)	1.23 (1.09–1.37)	13.4 (10.0–16.9)	1.22 (1.09–1.34)

Standardised to the distribution of income, education, civil status, year of diagnosis, sex, age, CCI, ASA, T and N stage and tumour location of the patients with severe mental illness.

95%CI: 95% Confidence interval; CSS: Cancer-specific survival; HR: Hazard Ratio; OS: Overall Survival; SAR: Survival after recurrence.

In the resection cohort, the standardised 5-year OS were 68 and 65% for those with mild and severe mental illness compared to 72% without mental illness. The HRs for OS comparing mild and severe mental disease to no mental disease were significantly increased at 1 and 3 years but decreased over time (Table 2, Figure 3). The median follow-up until censoring or death was 4.9 years (IQR 2.35–8.5).

The standardised 5-year CSS in the resection cohort were 77 and 74% for those with mild and severe mental illness compared to 79% without mental illness. The HRs for CSS comparing mild and severe mental disease to no mental disease were significantly increased at 1 and 3 years but decreased over time (Table 2, Figure 3). There was no association between the history of mental illness and TTR (Table 2, Figure 3).

## Survival after recurrence

**Figure 4:**
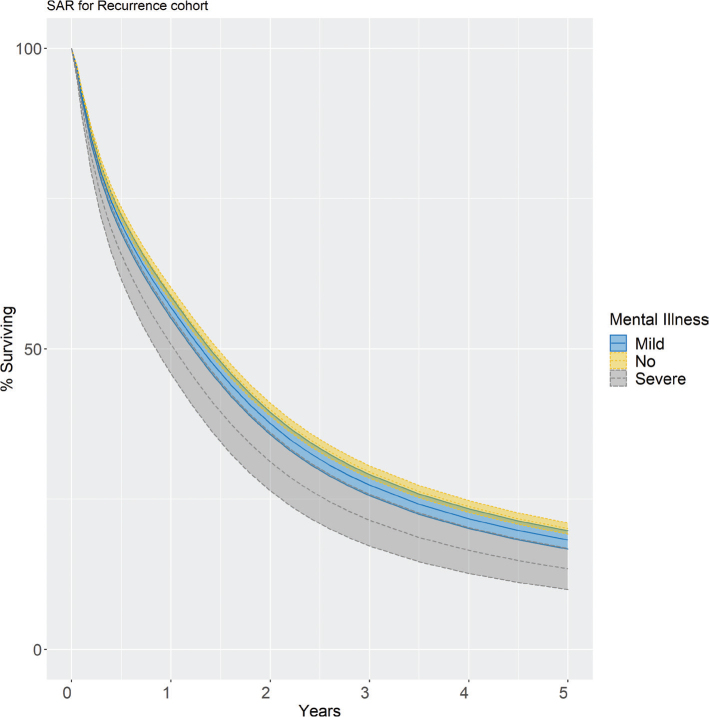
Standardised survival after recurrence for resected colorectal cancer patients with stage I-III disease by the history of mental illness. Standardised to the distribution of income, education, civil status, year of diagnosis, sex, age, CCI, ASA, T and N stage and tumour location of the patients with a history of severe mental illness. The shaded area corresponds to the 95% confidence interval.

SAR was analysed in 7,658 patients with recurrence, described in Supplementary Table 5. SAR was estimated after standardisation for income, education, civil status, sex, age, year of diagnosis and comorbidities, and the covariate pattern was set to that of the patients with severe mental illness. Modelling a time-varying effect for mental illness did not improve the model. The 1-year standardised SAR was 57 and 51% for those with mild and severe mental illness compared to 59% without mental illness (Table 2, Figure 4), corresponding to HRs of 1.06 (95%CI 1.01–1.11) and 1.24 (95% CI 1.09–1.39), respectively. The 3-year standardised SAR was 27 and 22% for those with mild and severe mental illness compared to 30% without mental illness. The median follow-up until censoring or death was 1.6 years (IQR 0.5–3.4).

## Sensitivity analysis adjusting for treatment

Models adjusted for emergency surgery and adjuvant treatment in the resected cohort were fitted to investigate if the observed survival differences in OS, CSS and TTR remained unchanged. There remained a statistically significant difference in OS and CSS for those with mental illness versus those without. There was no statistically significant difference in TTR (Supplementary Table 6 and Supplementary Figures 2–4).

## Discussion

This study aimed to investigate if there are differences in treatment and outcomes in stage I–III CRC dependent on the history of mental illness in a population-based cohort study of 69,633 CRC patients, and whether these differences remained after adjustment for pre-defined confounders, including detailed data on socioeconomic factors. There was no difference in the odds of cancer resection, but patients with a history of mental illness were more likely to have emergency surgery. Despite no statistically significant difference in multi-disciplinary team discussions, they were less likely to receive adjuvant treatment. Patients with a history of mental illness initially experienced an increased mortality rate, but after 5 years the elevated risk for overall mortality was only 10–11% and no increased cancer-specific mortality remained.

In line with previous studies, overall and CSS was worse in the patients with a history of mental illness [1, 8] Interestingly, CSS varied over time, where an increased risk of cancer death was observed during the first years but later decreased. A history of mental illnesses was not associated with worse TTR; however, following a recurrence prognosis was worse. The increased risk of CSS during the first 2 years after cancer resection may be driven by a greater proportion of emergency surgeries, a known risk factor for mortality [22]. A larger proportion of acute resections may be due to patients’ delay leading to more advanced tumours at presentation [22]. Patients with a history of mental illness were more deprived, which is associated with emergency surgery. However, despite adjustments patients with a history of severe mental illness had a 50% increased odds of emergency surgery. Patients with a history of mental illness had more somatic diseases and were more likely to experience postoperative complications, which may explain differences in the use of adjuvant therapy and differences in CSS. The base models adjusted for comorbidities and differences in OS and CSS remained when accounting for differences in emergency surgery and adjuvant treatment. Most recurrences occur early [23], and as there was no statistically significant difference in TTR, the worse prognosis could be explained by worse SAR.

The Swedish healthcare system is universal for all citizens and aims to provide equal access to care. The differences seen here are not unique to the healthcare system, with reports from the US describing worse CSS [1] and a lower chance of adjuvant therapy in line with the current study [1]. Advanced stage and lower odds of adjuvant treatment were also seen in Denmark [4] and France [5], and a Canadian cohort study showed that patients with mental illness had lower odds of receiving treatment recommended in guidelines [6]. From Finland, New Zealand and Japan there are reports of worse CSS in those with severe mental illness [3, 11, 13]. The Finish study reported HRs for CSS at 1.54–1.58 for patients from 1990 to 2013 with psychosis and 1.06–1.11 for those with mood-related disorders but did not adjust for SES [3]. HRs ranged from 1.25 to 1.89 in New Zealand for mild and severe mental illness, which corresponded to a 20–30% difference in survival at 5 years [11]. Similar differences were reported from Japan [13]. SAR was worse in the 16 patients with mental illness compared to those without in the Japanese study [13]. For the 1,645 patients with mental illness and recurrence in this paper, survival was worse than for those without a history of mental illness, especially in the 165 patients with severe mental illness. Differences in the treatment of recurrences and metastatic disease due to mental illnesses have yet to be investigated in Sweden, but there are reports of differences due to sex and hospital type [24, 25]. Most previous studies included fewer patients with a diagnosis before 2013, did not classify patients according to mental illness severity [2], reported survival as only HR or unadjusted rate.

## Strengths and weaknesses

The SCRCR is a rich data resource recently validated and covers at least 99% of all diagnosed CRC in Sweden [26, 27]. All patients who had surgery for a non-metastasized CRC were included in the analysis resulting in a large sample size reflective of practice in Sweden and generalizability to countries with a similar population and healthcare system. Personal identification numbers allow linkage of information from national registries and nearly complete follow-up. The COVID-19 pandemic should not have impacted the main results as the effects of the pandemic were limited [28, 29].

The study aimed to quantify the treatment and survival differences after adjusting for confounders. We could not investigate the presence of patients’ delay since data from general practitioners are not registered, nor information on how long or what symptoms patients had on presentation. Screening has been performed to a limited extent since 2008, but most county councils have not offered this in the studied period. Patients with a history of mental illness have a lower participation in screening which could lead to them being older at diagnosis, here we found the opposite, patients with a history of severe mental illness were younger than patients without mental illnesses. Emergency resection was more common in patients with a history of severe mental illness which may introduce lead time bias, this was mitigated by adjusting for stage in the main analysis and stage and emergency surgery in the sensitivity analysis. Comorbidities were assessed through the ASA classification and CCI which captures functional limitations and previous diagnoses.

Information on income and education was obtained from national registries, but some simplifications had to be made for our study as described previously [9]. Utilising the prescription registry allowed classification of patients with medicated mental illness. The coding of mental illness as mild and severe was chosen as practices may have changed over time, especially regarding how a disease is classified. The rationale for coding patients treated with anti-depressants without a contact with a psychiatric clinic as mild was that they were treated by a general practitioner’s office and that this would be at most of moderate severity. Diseases requiring treatment at a psychiatric clinic are severe, for example a diagnosis of schizophrenia, psychosis and bipolar disorder.

## Future perspective

Special care needs to be taken when dealing with patients with mental illness [30, 31], they need to be stable mentally to handle surgery, but as they are more likely to have emergency surgery this is a challenge. There are also challenges in providing information and enabling shared decision making as forced somatic care is not ethical nor legally possible. There is also the need to consider if the patient can make the number of visits required for oncological treatment, and what that will do to them from a mental health standpoint. Will they seek care if they have side effects? If not, treatment may be impossible. With all these factors it is not necessarily wrong that the results are what they are. Further research into differences in the treatment of metastatic disease is warranted based on the finding of substantial differences in SAR.

## Conclusion

Patients with a history of mental illnesses have more advanced tumours and are more likely to undergo emergency resection and less likely to receive adjuvant treatment. This impacts their prognosis, especially in the presence of recurrences. Clinicians should be aware of these differences so that the healthcare system can do what is possible to provide personalized care.

Compared to earlier studies from other countries the differences in Sweden were small, less than 10% at 5 years for OS and CSS. This suggests that these differences can be mitigated within a healthcare system characterized by universal access, standardised treatment pathways and mandatory multidisciplinary team conferences. While the results are encouraging, only time will tell if the differences can be eliminated completely.

## Supplementary Material

Mental illness and non-metastatic colorectal cancer treatment and survival, a nationwide study of almost 70,000 patients

## Data Availability

The data that support the findings of this study are available from the Swedish Colorectal Cancer Registry, Swedish National Board of Welfare and Statistics Sweden. Restrictions apply to the availability of these data, which were used under license for this study.
