# Supplementary material for: Mental illness and non-metastatic colorectal cancer treatment and survival, a nationwide study of almost 70,000 patients
Source: Acta Oncol. 2025 Apr 30;64:42710. doi: 10.2340/1651-226X.2025.42710 (PMC12053378; doi:10.2340/1651-226X.2025.42710)
Supplement: Mental illness and non-metastatic colorectal cancer treatment and survival, a nationwide study of almost 70,000 patients [file AO-64-42710-s1.pdf]

Supplementary material

Mental illness and non-metastatic colorectal cancer treatment and survival, a nationwide study of almost 70,000 patients

SUPPLEMENTARY TABLES 2

SUPPLEMENTARY TABLE 1: TUMOUR AND TREATMENT CHARACTERISTICS OF RESECTED COLORECTAL CANCER PATIENTS DIAGNOSED WITH STAGE I-III DISEASE IN SWEDEN DURING THE YEARS 2008-2021 BY THE HISTORY OF MENTAL ILLNESS (NO, MILD, SEVERE MENTAL ILLNESS) 2
SUPPLEMENTARY TABLE 2: DEMOGRAPHICS OF ALL STAGE I-III COLORECTAL PATIENTS REGARDLESS OF SURGERY OR NOT 3
SUPPLEMENTARY TABLE 3: ODDS RATIOS NO RESECTION, EMERGENCY SURGERY, MDT DISCUSSION, AND ONCOLOGICAL TREATMENT 4
SUPPLEMENTARY TABLE 4: LIKELIHOOD RATIO TEST X2AND P-VALUES FOR THE DIFFERENCE BETWEEN MODELS 5
SUPPLEMENTARY TABLE 5: DEMOGRAPHICS OF RESECTED COLORECTAL CANCER PATIENTS DIAGNOSED WITH RECURRENCE 6
SUPPLEMENTARY TABLE 6: PROPORTION SURVIVING AND HAZARD RATIOS FOR MODELS INCLUDING ADJUVANT TREATMENT AND EMERGENCY SURGERY 7

SUPPLEMENTARY FIGURES 8

SUPPLEMENTARY FIGURE 1: DIRECT ACYCLIC GRAPH OF RELATIONSHIP BETWEEN VARIABLES IN THE MAIN ANALYSIS FOR ADJUSTING THE DIRECT EFFECT OF MENTAL ILLNESS. 8
SUPPLEMENTARY FIGURE 2: STANDARDISED OVERALL SURVIVAL IN RESECTION COHORT BY THE HISTORY OF MENTAL ILLNESS 9
SUPPLEMENTARY FIGURE 3: STANDARDISED CANCER SPECIFIC SURVIVAL IN RESECTION COHORT BY THE HISTORY OF MENTAL ILLNESS 10
SUPPLEMENTARY FIGURE 4: STANDARDISED TIME TO RECURRENCE IN RESECTION COHORT BY THE HISTORY OF MENTAL ILLNESS 11

Supplementary Tables

Supplementary Table 1: Tumour and treatment characteristics of resected colorectal cancer patients diagnosed with stage I-III disease in Sweden during the years 2008-2021 by the history of mental illness (no, mild, severe mental illness)

Variables		Total	No	Mild mental illness	Severe mental illness
**Location**					
	Right	24,841 (41.4%)	18,710 (39.9%)	5,627 (47.7%)	504 (40.3%)
	Left	17,195 (28.7%)	13,500 (28.8%)	3,248 (27.5%)	447 (35.8%)
	Rectum	17,898 (29.8%)	14,683 (31.3%)	2,920 (24.7%)	295 (23.6%)
	Missing	61 (0.1%)	46 (0.1%)	11 (0.1%)	4 (0.3%)
**T**					
	T0-2	15,709 (26.2%)	12,176 (25.9%)	3,242 (27.5%)	291 (23.3%)
	T3	34,013 (56.7%)	26,820 (57.1%)	6,503 (55.1%)	690 (55.2%)
	T4	10,209 (17.0%)	7,895 (16.8%)	2,045 (17.3%)	269 (21.5%)
	Missing	64 (0.1%)	48 (0.1%)	16 (0.1%)	0 (0.0%)
**N**					
	N0	36,509 (60.9%)	28,495 (60.7%)	7,269 (61.6%)	745 (59.6%)
	N1	15,384 (25.6%)	11,982 (25.5%)	3,080 (26.1%)	322 (25.8%)
	N2	8,102 (13.5%)	6,462 (13.8%)	1,457 (12.3%)	183 (14.6%)
**Stage**					
	0	0 (0.0%)	0 (0.0%)	0 (0.0%)	0 (0.0%)
	1	12,897 (21.5%)	9,983 (21.3%)	2,674 (22.6%)	240 (19.2%)
	2	23,612 (39.4%)	18,512 (39.4%)	4,595 (38.9%)	505 (40.4%)
	3	23,486 (39.1%)	18,444 (39.3%)	4,537 (38.4%)	505 (40.4%)
	4	0 (0.0%)	0 (0.0%)	0 (0.0%)	0 (0.0%)
**Grade**					
	Low	45,909 (76.5%)	36,012 (76.7%)	8,951 (75.8%)	946 (75.7%)
	High	11,337 (18.9%)	8,710 (18.6%)	2,368 (20.1%)	259 (20.7%)
	Missing	2,749 (4.6%)	2,217 (4.7%)	487 (4.1%)	45 (3.6%)
**Vascular invasion**					
	No	40,267 (67.1%)	31,481 (67.1%)	8,014 (67.9%)	772 (61.8%)
	Yes	15,837 (26.4%)	12,145 (25.9%)	3,274 (27.7%)	418 (33.4%)
	Missing	3,891 (6.5%)	3,313 (7.1%)	518 (4.4%)	60 (4.8%)
**Perineural invasion**					
	No	44,144 (73.6%)	34,211 (72.9%)	9,007 (76.3%)	926 (74.1%)
	Yes	9,131 (15.2%)	7,039 (15.0%)	1,862 (15.8%)	230 (18.4%)
	Missing	6,720 (11.2%)	5,689 (12.1%)	937 (7.9%)	94 (7.5%)
**Mucinous**					
	No	47,311 (78.9%)	36,883 (78.6%)	9,406 (79.7%)	1,022 (81.8%)
	Yes	9,124 (15.2%)	7,133 (15.2%)	1,836 (15.6%)	155 (12.4%)
	Missing	3,560 (5.9%)	2,923 (6.2%)	564 (4.8%)	73 (5.8%)
**Neoadjuvant**					
	No	52,194 (87.0%)	40,483 (86.2%)	10,588 (89.7%)	1,123 (89.8%)
	Radiotherapy	6,394 (10.7%)	5,286 (11.3%)	1,006 (8.5%)	102 (8.2%)
	Radiochemo	760 (1.3%)	629 (1.3%)	119 (1.0%)	12 (1.0%)
	Chemotherapy	647 (1.1%)	541 (1.2%)	93 (0.8%)	13 (1.0%)
**Emergency Surgery**					
	Elective	53,248 (88.8%)	41,822 (89.1%)	10,395 (88.0%)	1,031 (82.5%)
	Emergency	6,717 (11.2%)	5,093 (10.9%)	1,405 (11.9%)	219 (17.5%)
	Missing	30 (0.1%)	24 (0.1%)	6 (0.1%)	0 (0.0%)
**Postoperative complications**					
	No	41,692 (69.5%)	32,865 (70.0%)	8,053 (68.2%)	774 (61.9%)
	Yes	18,010 (30.0%)	13,850 (29.5%)	3,692 (31.3%)	468 (37.4%)
	Missing	293 (0.5%)	224 (0.5%)	61 (0.5%)	8 (0.6%)
**Adjuvant**					
	No	47,420 (79.0%)	36,824 (78.5%)	9,568 (81.0%)	1,028 (82.2%)
	Chemotherapy	12,538 (20.9%)	10,084 (21.5%)	2,232 (18.9%)	222 (17.8%)
	Radiochemo	37 (0.1%)	31 (0.1%)	6 (0.1%)	0 (0.0%)

**TNM stage was classified as stage III if any T and N1-2M0, stage II if missing T and N0M0 or T3-4 and unknown N or N0 and unknown M or M0, and stage I if T1-2 and unknown N.*

Supplementary Table 2: Demographics of all stage I-III colorectal patients regardless of surgery or not in Sweden during the years 2008-2021 by the history of mental illness (No, mild, severe mental illness)

Variables	Total	No	Mild mental illness	Severe mental illness
**Sex**				
Male	36,446 (52.3%)	30,033 (55.6%)	5,616 (39.6%)	797 (54.2%)
Female	33,187 (47.7%)	23,966 (44.4%)	8,548 (60.4%)	673 (45.8%)
**Age (Years)**				
Median (IQR)	73.0 (65.0 - 80.0)	73.0 (65.0 - 80.0)	74.0 (66.0 - 81.0)	73.0 (65.0 - 80.0)
**ASA**				
1	8,519 (12.2%)	7,390 (13.7%)	1,025 (7.2%)	104 (7.1%)
2	31,999 (46.0%)	25,381 (47.0%)	6,018 (42.5%)	600 (40.8%)
3	18,350 (26.4%)	13,399 (24.8%)	4,456 (31.5%)	495 (33.7%)
4	1,699 (2.4%)	1,127 (2.1%)	511 (3.6%)	61 (4.1%)
5	35 (0.1%)	29 (0.1%)	5 (0.0%)	1 (0.1%)
Missing	9,031 (13.0%)	6,673 (12.4%)	2,149 (15.2%)	209 (14.2%)
**CCI**				
Median (IQR)	0.0 (0.0 - 2.0)	0.0 (0.0 - 2.0)	0.0 (0.0 - 2.0)	1.0 (0.0 - 2.0)
Missing	4,134 (5.9%)	4,134 (7.7%)	0 (0%)	0 (0%)
**Income**				
Q1	16,171 (23.2%)	11,956 (22.1%)	3,765 (26.6%)	450 (30.6%)
Q2	16,437 (23.6%)	12,234 (22.7%)	3,832 (27.1%)	371 (25.2%)
Q3	16,451 (23.6%)	12,652 (23.4%)	3,471 (24.5%)	328 (22.3%)
Q4	16,369 (23.5%)	12,958 (24.0%)	3,090 (21.8%)	321 (21.8%)
Missing	4,205 (6.0%)	4,199 (7.8%)	6 (0.0%)	0 (0.0%)
**Education**				
-9y	17,631 (25.3%)	13,518 (25.0%)	3,762 (26.6%)	351 (23.9%)
9y-12y	24,124 (34.6%)	17,864 (33.1%)	5,654 (39.9%)	606 (41.2%)
12y-	22,916 (32.9%)	17,809 (33.0%)	4,609 (32.5%)	498 (33.9%)
Missing	4,962 (7.1%)	4,808 (8.9%)	139 (1.0%)	15 (1.0%)
**Civil**				
Alone	30,750 (44.2%)	23,323 (43.2%)	6,718 (47.4%)	709 (48.2%)
Not alone	38,883 (55.8%)	30,676 (56.8%)	7,446 (52.6%)	761 (51.8%)
**Mental illness**				
None	53,999 (77.5%)	53,999 (100.0%)	0 (0.0%)	0 (0.0%)
Mild depression	14,164 (20.3%)	0 (0.0%)	14,164 (100.0%)	0 (0.0%)
Severe depression	19 (0.0%)	0 (0.0%)	0 (0.0%)	19 (1.3%)
Psychosis	372 (0.5%)	0 (0.0%)	0 (0.0%)	372 (25.3%)
Bipolar	1,079 (1.5%)	0 (0.0%)	0 (0.0%)	1,079 (73.4%)
**Location**				
Right	27,036 (38.8%)	20,199 (37.4%)	6,271 (44.3%)	566 (38.5%)
Left	20,086 (28.8%)	15,675 (29.0%)	3,912 (27.6%)	499 (33.9%)
Rectum	22,333 (32.1%)	18,003 (33.3%)	3,932 (27.8%)	398 (27.1%)
Missing	178 (0.3%)	122 (0.2%)	49 (0.3%)	7 (0.5%)
**T**				
T0-2	18,133 (26.0%)	14,053 (26.0%)	3,750 (26.5%)	330 (22.4%)
T3	34,230 (49.2%)	26,996 (50.0%)	6,541 (46.2%)	693 (47.1%)
T4	10,489 (15.1%)	8,111 (15.0%)	2,100 (14.8%)	278 (18.9%)
Missing	6,781 (9.7%)	4,839 (9.0%)	1,773 (12.5%)	169 (11.5%)
**N**				
N0	37,819 (54.3%)	29,516 (54.7%)	7,540 (53.2%)	763 (51.9%)
N1	15,478 (22.2%)	12,058 (22.3%)	3,096 (21.9%)	324 (22.0%)
N2	8,188 (11.8%)	6,526 (12.1%)	1,478 (10.4%)	184 (12.5%)
Missing	8,148 (11.7%)	5,899 (10.9%)	2,050 (14.5%)	199 (13.5%)
**c/pStage**				
0	0 (0.0%)	0 (0.0%)	0 (0.0%)	0 (0.0%)
1	14,921 (21.4%)	11,458 (21.2%)	3,180 (22.5%)	283 (19.3%)
2	24,844 (35.7%)	19,397 (35.9%)	4,916 (34.7%)	531 (36.1%)
3	25,725 (36.9%)	20,073 (37.2%)	5,090 (35.9%)	562 (38.2%)
4	0 (0.0%)	0 (0.0%)	0 (0.0%)	0 (0.0%)
Missing	4,143 (5.9%)	3,071 (5.7%)	978 (6.9%)	94 (6.4%)
**Emergency Surgery**				
Elective	54,802 (78.7%)	43,019 (79.7%)	10,725 (75.7%)	1,058 (72.0%)
Emergency	7,074 (10.2%)	5,350 (9.9%)	1,492 (10.5%)	232 (15.8%)
Missing/No surgery	7,757 (11.1%)	5,630 (10.4%)	1,947 (13.7%)	180 (12.2%)

*TNM stage was classified as stage III if any T and N1-2M0, stage II if missing T and N0M0 or T3-4 and unknown N or N0 and unknown M or M0, and stage I if T1-2 and unknown N

ASA: American Society of Anesthesiologists classification

CCI: Charlson Comorbidity Index

IQR: Interquartile range

Q: Quartile

Supplementary Table 3: Odds ratios no resection, emergency surgery, MDT discussion, and oncological treatment by the history of mental illness (No, mild, severe mental illness)

Outcome	Mental illness status	OR (95%CI)	N
**No resection**	***Mild***	1.10 (0.94-1.28)	57137
	Severe	0.96 (0.62-1.50)	
**Emergency surgery**	***Mild***	1.03 (0.96-1.10)	54505
	Severe	1.56 (1.32-1.84)	
**Preoperative MDT**	***Mild***	0.90 (0.83-0.98)	48429
	Severe	0.86 (0.68-1.08)	
**Postoperative MDT**	***Mild***	0.97 (0.89-1.05)	53317
	Severe	0.95 (0.75-1.19)	
**Neoadjuvant treatment (rectal cancers)**	***Mild***	0.92 (0.85-1.01)	16159
	Severe	0.96 (0.75-1.22)	
**Adjuvant treatment (colon cancers)**	***Mild***	0.84 (0.78-0.91)	37839
	Severe	0.65 (0.53-0.80)	

CI: Confidence interval
OR: Odds ratio

Supplementary Table 4: Likelihood ratio test χ^2 and p-values for the difference between models with and without exposure and time-varying effect

Outcome	Covariates only vs Covariates and exposure p	Covariates and Exposure vs TVE p	Base model N	TVE vs Expanded p	Expanded model N
**OS (all)**	<0·001	<0·001	60,795		
**OS (op)**	<0·001	<0·001	41,386	<0·001	41,373
**CSS**	<0·001	<0·001	41,386	<0·001	41,373
**TTR**	0·06	0·78	41,386	<0·001	41,373
**SAR**	<0·001	0·25	7,658		

CSS: Cancer-specific survival

OS: Overall survival, all patients or only operated patients

SAR: Survival after recurrence

TTR: Time to recurrence

TVE: Time-varying effect for exposure and covariates

Expanded model: TVE and emergency surgery and adjuvant treatment

Supplementary Table 5: Demographics of resected colorectal cancer patients diagnosed with recurrence after stage I-III disease in Sweden during the years 2008-2021 by the history of mental illness (No, mild, severe mental illness)

Variables	Total	No	Mild mental illness	Severe mental illness
**Sex**				
Male	4,745 (54.8%)	4,023 (57.4%)	631 (42.6%)	91 (55.2%)
Female	3,912 (45.2%)	2,989 (42.6%)	849 (57.4%)	74 (44.8%)
**Age (Years)**				
Median (IQR)	71.0 (64.0 - 78.0)	71.0 (64.0 - 78.0)	71.0 (63.0 - 79.0)	72.0 (63.0 - 80.0)
**ASA**				
1	1,373 (15.9%)	1,223 (17.4%)	139 (9.4%)	11 (6.7%)
2	4,561 (52.7%)	3,710 (52.9%)	764 (51.6%)	87 (52.7%)
3	2,341 (27.0%)	1,772 (25.3%)	510 (34.5%)	59 (35.8%)
4	176 (2.0%)	121 (1.7%)	51 (3.4%)	4 (2.4%)
5	3 (0.0%)	3 (0.0%)	0 (0.0%)	0 (0.0%)
Missing	203 (2.3%)	183 (2.6%)	16 (1.1%)	4 (2.4%)
**CCI**				
Median (IQR)	0.0 (0.0 - 2.0)	0.0 (0.0 - 1.0)	0.0 (0.0 - 2.0)	0.0 (0.0 - 2.0)
Missing	743 (8.6%)	743 (10.6%)	0 (0%)	0 (0%)
**Income**				
Q1	2,028 (23.4%)	1,543 (22.0%)	430 (29.1%)	55 (33.3%)
Q2	2,026 (23.4%)	1,597 (22.8%)	393 (26.6%)	36 (21.8%)
Q3	1,983 (22.9%)	1,586 (22.6%)	356 (24.1%)	41 (24.8%)
Q4	1,868 (21.6%)	1,536 (21.9%)	299 (20.2%)	33 (20.0%)
Missing	752 (8.7%)	750 (10.7%)	2 (0.1%)	0 (0.0%)
**Education**				
-9y	2,079 (24.0%)	1,699 (24.2%)	338 (22.8%)	42 (25.5%)
9y-12y	2,939 (33.9%)	2,243 (32.0%)	633 (42.8%)	63 (38.2%)
12y-	2,798 (32.3%)	2,245 (32.0%)	493 (33.3%)	60 (36.4%)
Missing	841 (9.7%)	825 (11.8%)	16 (1.1%)	0 (0.0%)
**Civil**				
Alone	3,747 (43.3%)	3,027 (43.2%)	635 (42.9%)	85 (51.5%)
Not alone	4,910 (56.7%)	3,985 (56.8%)	845 (57.1%)	80 (48.5%)
**Metastatic Location**				
Liver only	2,322 (26.8%)	1,894 (27.0%)	386 (26.1%)	42 (25.5%)
Lung only	1,835 (21.2%)	1,492 (21.3%)	319 (21.6%)	24 (14.5%)
Liver and Lung	1,295 (15.0%)	1,048 (14.9%)	213 (14.4%)	34 (20.6%)
Peritoneal	81 (0.9%)	57 (0.8%)	23 (1.6%)	1 (0.6%)
Local only	1,177 (13.6%)	961 (13.7%)	195 (13.2%)	21 (12.7%)
Other only	12 (0.1%)	8 (0.1%)	4 (0.3%)	0 (0.0%)
Multiple	863 (10.0%)	697 (9.9%)	151 (10.2%)	15 (9.1%)
Unknown	1,048 (12.1%)	836 (11.9%)	184 (12.4%)	28 (17.0%)
Missing	24 (0.3%)	19 (0.3%)	5 (0.3%)	0 (0.0%)

*TNM stage was classified as stage III if any T and N1-2M0, stage II if missing T and N0M0 or T3-4 and unknown N or N0 and unknown M or M0, and stage I if T1-2 and unknown N

ASA: American Society of Anesthesiologists classification

CCI: Charlson Comorbidity Index

IQR: Interquartile range

Q: Quartile

Supplementary Table 6: Proportion surviving and Hazard ratios for models including adjuvant treatment and emergency surgery for Overall survival in resected cohort, Cancer-specific survival (CSS) and Time to recurrence (TTR) by the history of mental illness(No, mild, severe mental illness) at 1, 3 and 5 years for resected colorectal cancer patients diagnosed with stage I-III disease in Sweden during the years 2008-2021.

Outcome	Mental Illness	1-year Survival % (95%CI)	1-year HR (95%CI)	3-year Survival % (95%CI)	3-year HR (95%CI)	5-year Survival % (95%CI)	5-year HR (95%CI)
**OS op**	***No***	95.0 (94.8-95.2)		81.6 (81.2-82.0)		70.9 (70.5-71.4)	
	Mild	93.6 (93.2-94.1)	1.18 (1.11-1.24)	78.6 (77.9-79.4)	1.11 (1.07-1.16)	67.2 (66.4-68.0)	1.12 (1.08-1.17)
	Severe	91.9 (90.5-93.3)	1.50 (1.31-1.69)	75.0 (72.8-77.2)	1.14 (1.01-1.27)	64.4 (62.1-66.8)	0.96 (0.80-1.12)
**CSS**	***No***	96.1 (95.9-96.3)		85.5 (85.2-85.9)		77.9 (77.5-78.4)	
	Mild	95.1 (94.7-95.5)	1.14 (1.06-1.22)	83.7 (83-84.4)	1.06 (1.00-1.11)	75.8 (74.9-76.6)	1.05 (0.98-1.12)
	Severe	93.8 (92.6-95.1)	1.39 (1.17-1.61)	81.3 (79.1-83.4)	1.06 (0.9-1.22)	73.9 (71.4-76.3)	0.89 (0.66-1.12)
**TTR**	***No***	91.6 (91.3-91.9)		80.8 (80.3-81.2)		78.5 (78.0-79.0)	
	Mild	91.3 (90.9-91.8)	1.04 (0.98-1.09)	80.1 (79.3-80.9)	1.03 (0.99-1.08)	77.8 (76.9-78.7)	1.03 (0.99-1.08)
	Severe	91.2 (90.0-92.4)	1.05 (0.91-1.19)	79.9 (77.5-82.3)	1.04 (0.92-1.17)	77.6 (75.0-80.2)	1.04 (0.92-1.16)

Standardised to the distribution of income, education, civil status, year of diagnosis, sex, age, CCI, ASA, T and N stage and tumour location, adjuvant treatment and emergency surgery of the patients with severe mental illness
95%CI: 95% Confidence interval
CSS: Cancer-specific survival
HR: Hazard Ratio
OS: Overall Survival

Supplementary figures

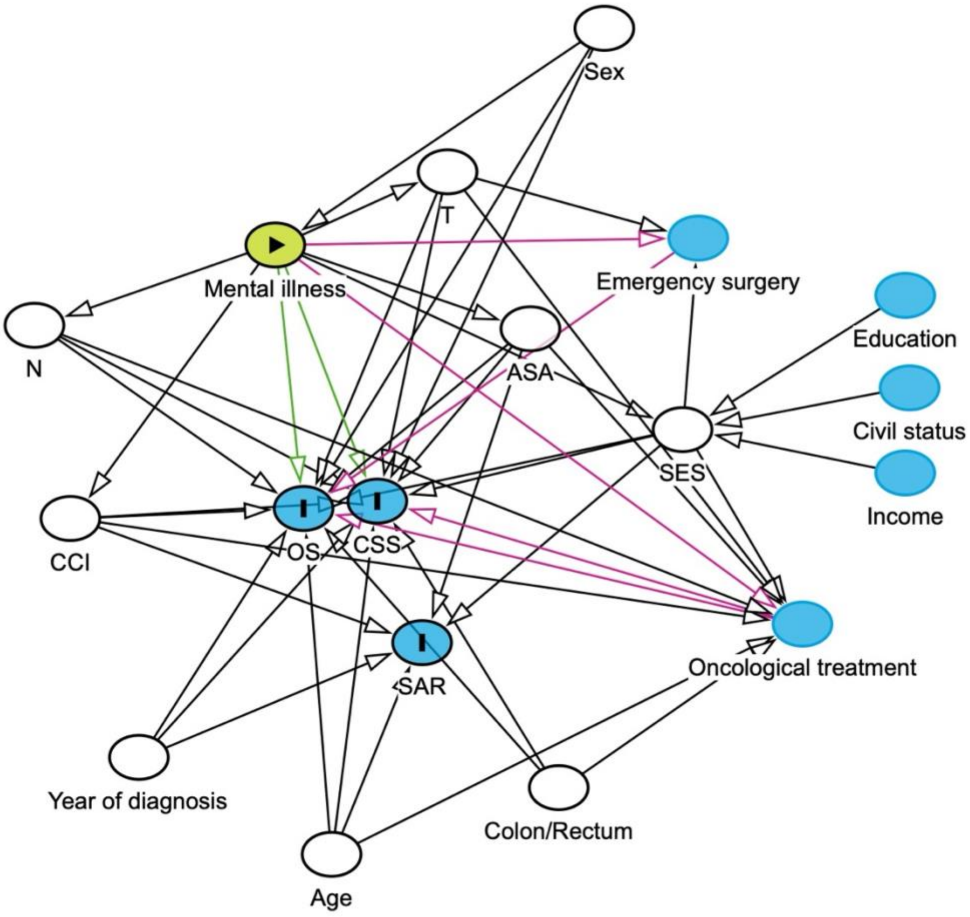

Supplementary Figure 1: Direct Acyclic graph of relationship between variables in the main analysis for adjusting the direct effect of Mental illness.

Black line: Correctly adjusted. Red line: Not adjusted but should be for direct effect estimation (and was adjusted for in sensitivity analysis), Green: Exposure, Blue with line: Outcomes, Blue: Confounders, SES: To minimise arrows Income, Civil status and education is represented by SES.

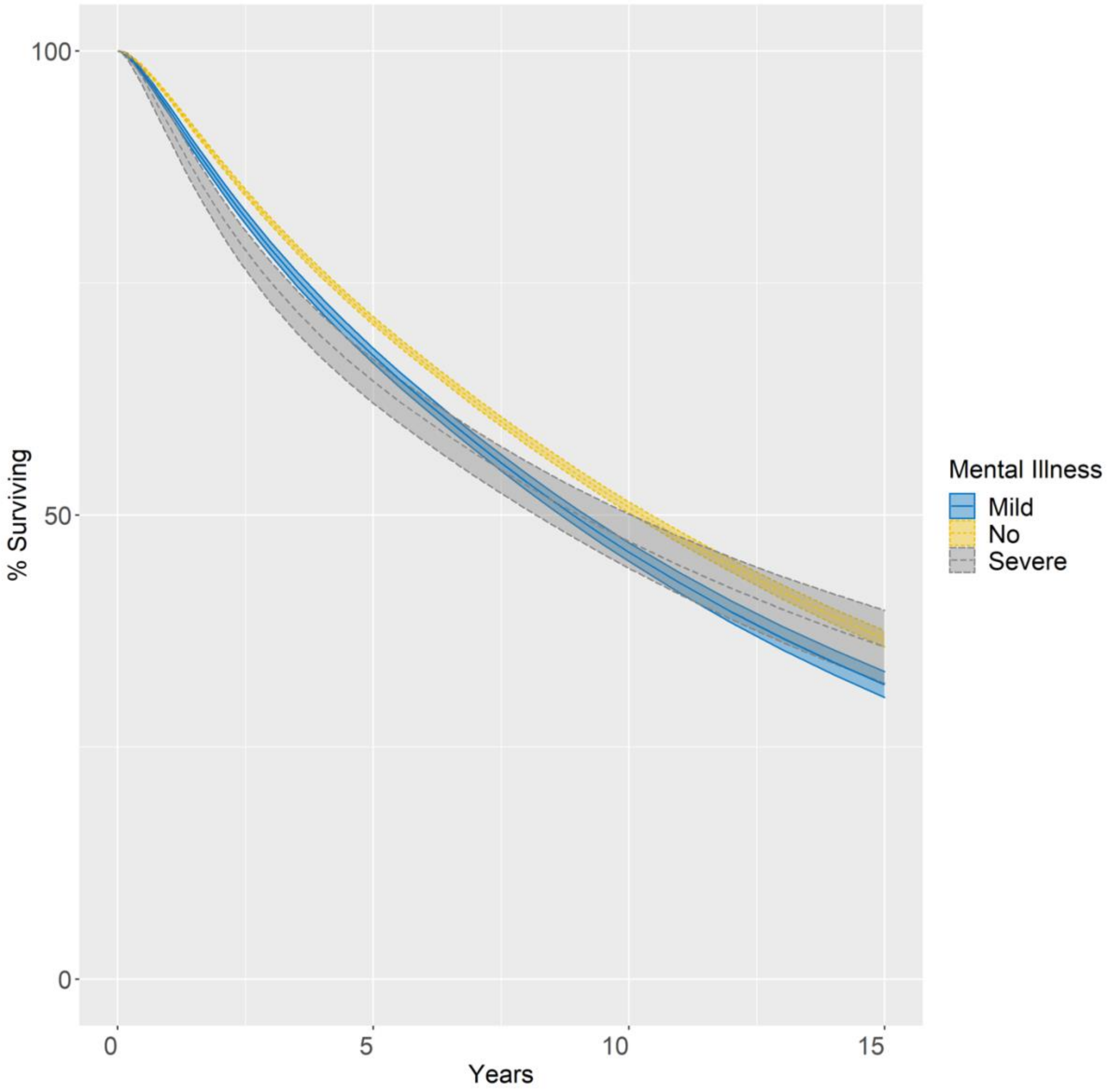

Supplementary Figure 2: Standardised overall survival in resection cohort by the history of mental illness. Standardised to the distribution of income, education, civil status, year of diagnosis, sex, age, CCI, ASA, T and N stage and tumour location, emergency surgery and adjuvant treatment of the patients with a history of severe mental illness. The shaded area corresponds to the 95% confidence interval.

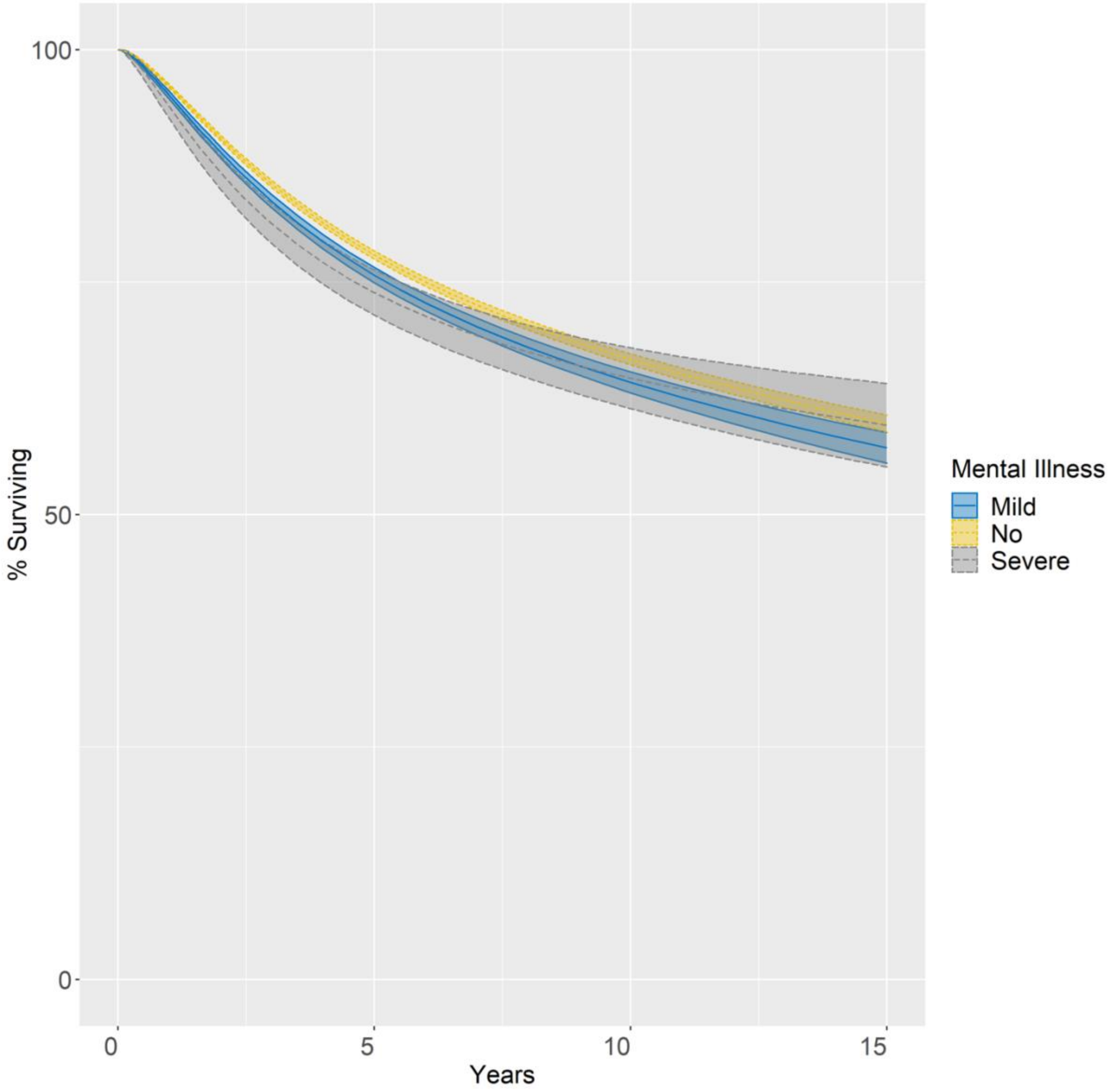

Supplementary Figure 3: Standardised cancer specific survival in resection cohort by the history of mental illness. Standardised to the distribution of income, education, civil status, year of diagnosis, sex, age, CCI, ASA, T and N stage and tumour location, emergency surgery and adjuvant treatment of the patients with a history of severe mental illness. The shaded area corresponds to the 95% confidence interval.

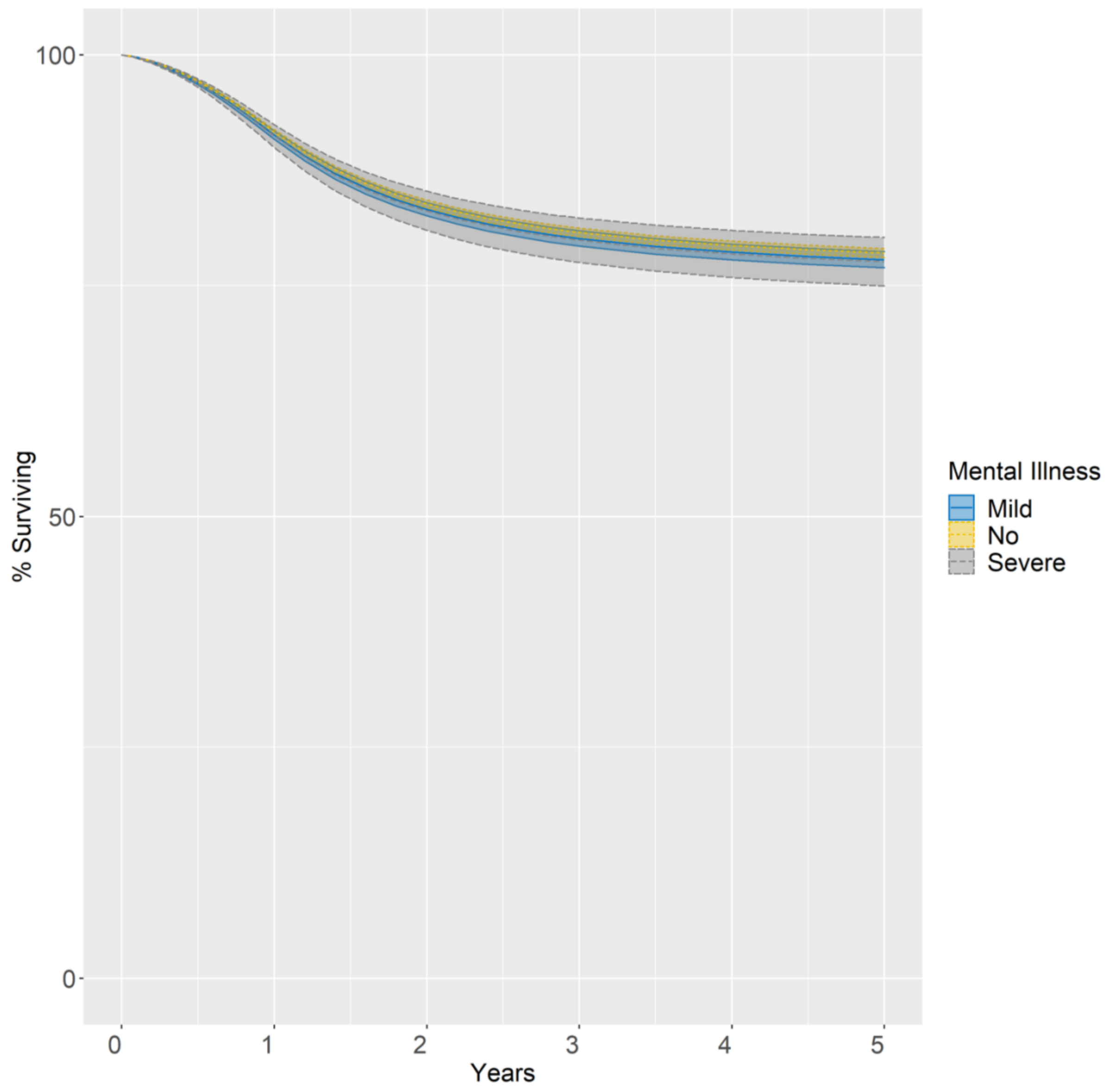

Supplementary Figure 4: Standardised time to recurrence in resection cohort by the history of mental illness. Standardised to the distribution of income, education, civil status, year of diagnosis, sex, age, CCI, ASA, T and N stage and tumour location, emergency surgery and adjuvant treatment of the patients with a history of severe mental illness. The shaded area corresponds to the 95% confidence interval.
